# Alterations in skin microbiome mediated by radiotherapy and their potential roles in the prognosis of radiotherapy-induced dermatitis: a pilot study

**DOI:** 10.1038/s41598-021-84529-7

**Published:** 2021-03-04

**Authors:** Mohammed Ramadan, Helal F. Hetta, Moustafa M. Saleh, Mohamed E. Ali, Ali Aya Ahmed, Mohammed Salah

**Affiliations:** 1 Microbiology and Immunology Department, Faculty of Pharmacy, Al-Azhar University-Assiut Branch, Assiut, 71526 Egypt; 2 Department of Medical Microbiology and Immunology, Faculty of Medicine, Assiut University, Assiut, 71515 Egypt; 3 Department of Internal Medicine, University of Cincinnati College of Medicine, Cincinnati, OH 45267-0595 USA; 4 Microbiology and Immunology Department, Faculty of Pharmacy Port, Said University, Port Said, 42526 Egypt; 5 Microbiology and Immunology Department, Faculty of Pharmacy, Sinai University, Ismaillia, 41611 Egypt

**Keywords:** Microbiology, Pathogenesis

## Abstract

Radiotherapy-induced dermatitis (RID) is an inflammatory cutaneous disorder that is acquired as an adverse effect of undergoing radiotherapy. Skin microbiome dysbiosis has been linked to the outcomes of several dermatological diseases. To explore the skin microbiota of RID and deduce their underlying impact on the outcome of RID, cutaneous microbiomes of 78 RID patients and 20 healthy subjects were characterized by sequencing V1-V3 regions of 16S rRNA gene. In total, a significantly apparent reduction in bacterial diversity was detected in microbiomes of RID in comparison to controls. Overall, the raised Proteobacteria/ Firmicutes ratio was significantly linked to delayed recovery or tendency toward the permanence of RID (Kruskal Wallis: *P* = 2.66 × 10^–4^). Moreover, applying enterotyping on our samples stratified microbiomes into A, B, and C dermotypes. Dermotype C included overrepresentation of *Pseudomonas, Staphylococcus* and *Stenotrophomonas* and was markedly associated with delayed healing of RID. Strikingly, coexistence of diabetes mellitus and RID was remarkably correlated with a significant overrepresentation of *Klebsiella* or *Pseudomonas* and *Staphylococcus*. Metabolic abilities of skin microbiome could support their potential roles in the pathogenesis of RID. Cutaneous microbiome profiling at the early stages of RID could be indicative of prospective clinical outcomes and maybe a helpful guide for personalized therapy.

## Introduction

Worldwide, there is a raised incidence of cancer^1^. Recently, Egypt recorded an exponentially increased rate of cancer patients. Radiotherapy is substantially considered as a key element of treatment in breast cancer and nearly is applied to > 70% of cancer patients^2–5^.

Although the rapid advances in radiation oncology improved the effectiveness of cancer treatment, skin reactions, which are defined as radiation dermatitis, remain the most common adverse effect of radiation toxicity^6^. Overall, cutaneous dermatitis negatively affects individuals’ health and quality of life. Pathophysiology of cutaneous dermatitis was linked to a multitude of predisposing factors that could cause a disturbance in proinflammatory and profibrotic cytokines^7,8^. These factors include genetic and epigenetic predisposition in addition to arterial insufficiency, diabetes mellitus, chronic osteomyelitis, radiotherapy-induced dermatitis (RID), rheumatoid arthritis, skin tumors, trauma and venous ulcers^1,9,10^. Distinctly, the vast majority of cancer patients experience skin reactions upon radiation exposure. Several risk factors are assumed to directly trigger or influence the incidence and severity of RID. These risk factors may be intrinsic including age, ethnicity, gender, malnutrition, location and stage of the tumor, and concomitant diseases such as systemic inflammation and diabetes mellitus. While extrinsic factors include the kind and total dose of treatment, volume of treated area and radiotherapy technique^11^, in addition to bacterial infections^12^.

The anatomical and physiological structures of human skin represent the protective shield of the human body. The human skin is influenced by the surroundings and is the site where billions of microbial communities collectively constitute a unique ecosystem that constantly modulates host immunity and metabolism. The normal functions of human skin basically require integral collaborations between epidermal barriers, skin immunity and microbial inhabitants. Both the composition and structure of the microbial ecosystems associated with the human skin are influenced by several factors which include host-related such as age and gender, in addition to external stimuli as weather, lifestyle, and use of medicated preparations^13,14^.

Despite the skin microbiomes have been intensely investigated over the past years for their implication in several dermatological disorders, their roles in the pathogenesis and prognosis of RID demand comprehensive investigations. We aimed to provide new insight into the potential impact of skin microbiome on the prognosis of RID.

## Methods and materials

### Ethics statement

The present study was approved by the ethics committee of the Faculty of Pharmacy, Port Said University, Egypt (Reference no. A-6-2019). This study was carried out following the principles of the Declaration of Helsinki. Recruited patients in this study provided informed consent at sampling time.

### Study design and recruitment of participants

This study was performed for over 16 months (between June 2019 and September 2020). Participants were prospectively enrolled at outpatient clinics of dermatology department, Al Mabarrah Health Insurance Hospital, Zagazig, Egypt. The prognosis of RID was identified by follow-up of patients for 12 months. Diagnosis and grading of RID were carried out according to CDC Cutaneous Radiation Injury: Fact Sheet for Physicians^15,16^. The inclusion criterion was mainly based on patients with newly diagnosed and grade 2 confirmed RID. The exclusion criteria were: (1) systemic antibiotic intake; (2) topical application of corticosteroids and antibiotics. Besides, healthy subjects were involved in the current study. All recruited patients provided informed consent prior to sampling.

### Sample collection, DNA extraction and sequencing of 16S rRNA gene

Skin swabs were collected from patients by rubbing the site of RID with a premoistened swab (0.15 M NaCl with 0.1%Tween 20). Also, antecubital fossa, represents the moist sites, was rubbed by swab for collecting specimens from healthy subjects^17^. The collected swabs were used for genomic bacterial DNA extraction using DNeasy PowerSoil Kit Catalog No. 12888 (Qiagen, Valencia, USA) with previously reported modifications^18^. V1-V3 hypervariable regions of 16S rRNA gene were amplified using the following primers (underlined bases define adaptors): Forward Primer 5′ AATGATACGGCGACCACCGAGATCTACACATCGTACGTATGGTAATTC AATTACCGCGGCT GCTGG; Reverse Primer 5′ CAAGCAGAAGACGGCATACGAGATAACTCTCGAG TCAGTCA GCCGAGTTTGATCMTGGCTCAG^19^. Then, PCR amplicons were sequenced by Illumina MiSeq at IGA Technology Services (Udine, Italy).

### Bioinformatic analyses of row reads of 16S rRNA

The analysis and classification of 16S rRNA reads were mainly based on ribosomal sequence variants (RSV). QIIME2 platform with DADA2 plugin was used for sequence preprocessing^20^, which included: trimming, filtering and denoising of row reads as previously described (maxEE = 2; truncation length for forward and reverse reads 270 and 210, respectively; Phred quality ≥ 25)^21^. Finally, high-resolution RSVs were generated by removing the potential chimeric sequences.

RDP’s naive Bayesian classifier was applied on representative RSVs for taxonomy assignment against SILVA SSU Ref NR dataset v.132 at 99% sequence similarity^22,23^.

Bacterial diversity was estimated using QIIME2 on the entire dataset without subsampling. Alpha diversity was determined using two approaches; regarding richness (number of observed species and Chao1 index) and evenness of communities (Shannon diversity index). The statistical significance of pairwise comparisons was assessed using unpaired Wilcoxon rank sum test, while multiple group comparisons were estimated using Kruskal–Wallis rank sum test and all P values were corrected using false discovery rate method (FDR)^24^. The core genus in our dataset was defined as the genus that was detected in 80% of all samples, while the core genus for each study group was defined as the genus that was present in all samples of this group.

Phylogenetic relatedness of bacterial communities was identified using both unweighted and weighted UniFrac matrices^25^. Similarity distances between cutaneous microbiomes were visualized by principal coordinates analysis (PCoA) based on weighted UniFrac distance matrix. Permutational Multivariate Analysis of Variance (Adonis R, package Vegan) was applied to define the significance of weighted UniFrac distance-based clustering of samples^26^.

To determine the dermotypes of cutaneous microbiomes, a widely used enterotyping pipeline was applied to all genera in our dataset. Clustering of samples and the optimum number of clusters were achieved using partitioning around medoids (PAM) algorithm, Jensen–Shannon divergence (JSD) and Calinski-Harabasz (CH) index^27^.

For predicting the functional profile of cutaneous microbiota, representative RSVs were employed for further taxonomic classification using UCLUST^28^ against Greengenes database (V 13.8)^29^ at a 97% identity using closed-reference script for OTU picking in QIIME, v 1.9.1^29^. Functional potential of cutaneous microbiomes was predicted using Phylogenetic Investigation of Communities by Reconstruction of Unobserved States (PICRUSt) for mapping the Kyoto Encyclopedia of Genes and Genome (KEGG) Orthology (KO) Database at level 3^30–32^. LEfSe was applied to the metagenomic data to define the taxon or metabolic pathway (logarithmic LDA scores > 3.0 and α = 0.05) that were differentially abundant among healthy subjects and patients with RID (https://huttenhower.sph.harvard.edu/lefse/)^33^.

Correlations between either communities’ members or structure of microbiota and patient’s clinical data were defined by Spearman correlation coefficient (r ≥  ± 0.6, *P* ≤ 0. 01) using R package, Hamsic^34^. R packages; Phyloseq and ggpubr, were applied for calculation and plotting of diversity indices (https://rpkgs.datanovia.com/ggpubr/)^35,36^, while ggplot2 was used for graphical illustrations of results (http://userweb.eng.gla.ac.uk/umer.ijaz/bioinformatics/ecological.html)^37^.

### Data availability

16S rRNA row reads of this study have been deposited in NCBI bioproject under accession number PRJNA665254 (http://www.ncbi.nlm.nih.gov/bioproject/665254) and biosamples (SAMN168624511: SAMN16862528).

## Results

### Patient’s characteristics

Ninety-eight subjects were included in this study (20 controls and 78 patients with RID). Patients’ data regarding clinical characteristics, demography and outcome were listed in Table 1 (Supplemental Table S1).

**Table 1:** Summary of demographic data and clinical characteristics of the individuals enrolled.

	Patients with RID (N = 78) N (%)	Control group (N = 20) N (%)
Age(mean ± SD)	49.42 ± 8.79 years	36.35 ± 13.46 years
**Sex**		
Male	35 (44.9%)	10(50%)
Female	43 (55.1%)	10(50%)
**Cancer type**		NA
Breast	27 (34.6%)	
Lung	13 (16.7%)	
Colorectal	11 (14.1%)	
Prostate	9 (11.5%)	
Brain	5 (6.4%)	
Cervical	6 (7.6%)	
Oropharyngeal	4 (5.1%)	
Lymphoma	3 (3.8%)	
**Concomitant diseases**		NA
Diabetes miletus	36(46.1%)	
Hypertension	21(26.9%)	
**Outcome**		NA
Recovery after 2 weeks	12(15.4%)	
Recovery after 3 weeks	16(20.5%)	
Recovery after 4 weeks	5(6.4%)	
Recovery after 5 weeks	7(9%)	
Recovery after 6 weeks	7(9%)	
Recovery after 7 weeks	10(12.8%)	
Chronic ulcer	21(26.9%)	

*N* number of subjects, *NA* not applicable, *SD* standard deviation, % = percentage of all samples.

### Preprocessing and analyses of 16S rRNA sequences

In total, 11,276,243 row sequences (average reads per sample = 11,564) were inputted in Qiime2 for merging, quality checking, removal of low quality reads (1,405,020 sequences, 12.46% of all dataset) and potential chimeric sequences (587,492 sequences, 5.21% of all dataset) that were resulted in 9,283,731high quality sequences that were used for downstream analyses.

### Distinct taxonomic profile of cutaneous microbiomes related to RID

**Figure 1:**
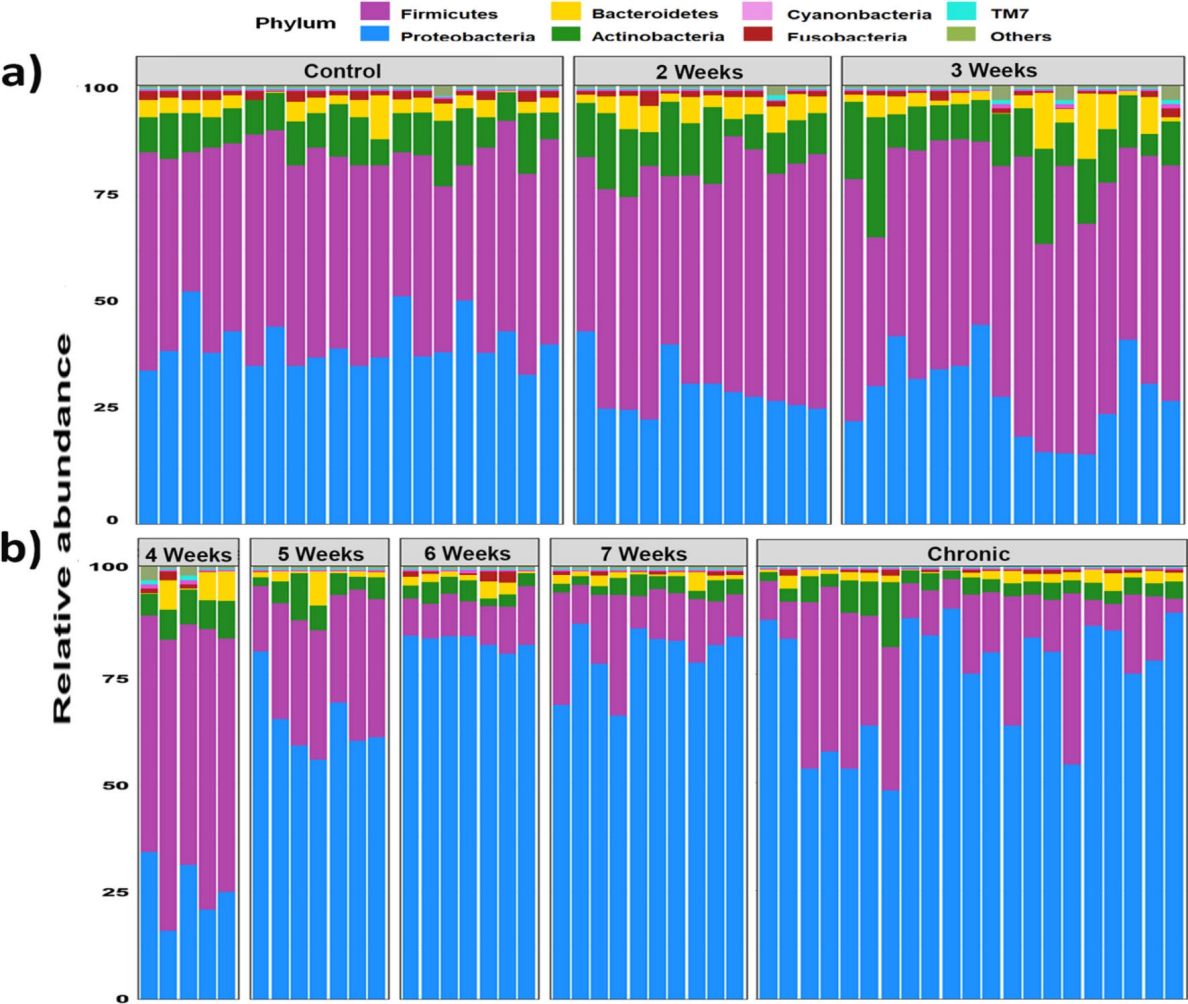
Phylum level analysis of cutaneous microbiota. Bar charts illustrate the relative proportions of the dominant phyla in skin microbiome of all studied groups. (**a**) Cutaneous microbiomes of rapidly recovered RID. (**b**) Cutaneous microbiomes of RID that showed delayed healing or turned to chronic ulcer. X-axis denotes the microbiome profile of each enrolled subjects, while Y-axis indicates the relative abundance of each phyla^37^.

16S rRNA reads were taxonomically assigned to 19 phyla, 58 classes, 109 orders, 237 families, 658 genera and 4643 OTUs. In total, cutaneous microbiomes associated to RID showed a remarkable representation of certain bacterial taxa at different taxonomic levels that was in contrast to healthy subjects (Fig. 1). Phylum level analyses of RID microbiomes exhibited alternately significant predominance of Firmicutes and Proteobacteria (Mean relative abundance ± SD: 55.39 ± 25.07% and 32.45 ± 19.79%, respectively) in addition to relatively constant proportions of Actinobacteria (Mean relative abundance ± SD: 7.15 ± 5.18%). Classification of RID samples to seven groups, regarding the time required for recovery of RID, revealed significant representation and coexistence of Proteobacteria and Firmicutes (Kruskal Wallis; *P* = 2.58 × 10^–7^: Spearman; *r* = -0.68, *P* = 0.001) (Supplemental Table S2). Microbiomes associated to rapidly healed RID (2, 3, 4 weeks) were significantly accompanied by the predominance of Firmicutes over Proteobacteria (Mean relative abundance: 52.2% and 29.42%, respectively; Kruskal Wallis: *P* = 5.71 × 10^–4^). In contrast, microbiomes of chronic ulcers were significantly predominated by Proteobacteria and low abundance of Firmicutes (Mean relative abundance: 74.01% and 18.36%, respectively; Kruskal Wallis, *P* = 0.00083).

### Reduced bacterial diversity characterize the shifts in cutaneous microbiomes of RID

**Figure 2:**
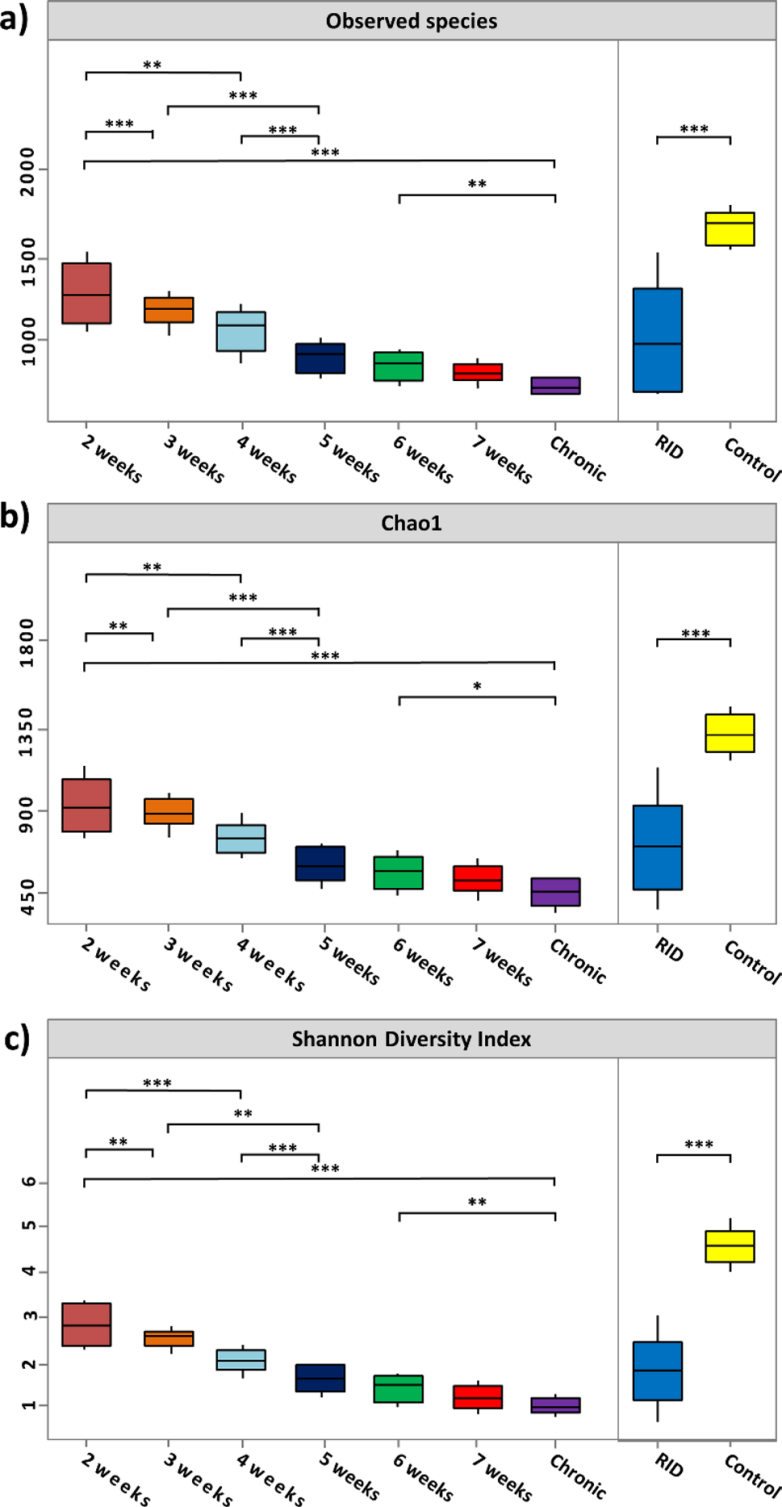
Alpha diversity of cutaneous microbiome was represented by box plots of the following indices: (**a**) the number of observed species and (**b**) Chao1 index, and (**c**) Shannon diversity index. Study groups were plotted on the X-axis and alpha indices were plotted on the Y-axis. The median is defined by the line in each box, the interquartile range (IQR) between the 25th and 75th percentile were delimited by the box, and the range was represented by the whisker. Statistical significance of pairwise comparisons was defined using the nonparametric Wilcoxon rank-sum test. Only significant differences were displayed with either * (p < 0.05), ** (p < 0.01) or *** (p < 0.001)^36^.

Identification of bacterial diversity associated with RID was performed using both evenness and richness indices. Skin microbiomes of healthy subjects were significantly more diverse than those with RID (Wilcoxon test: *P* = 0011.47 × 10–5) (Fig. 2). Surprisingly, cutaneous microbiomes of RID showed a significant reduction in bacterial diversity in conjugation with a prolonged duration of RID (Kruskal Wallis, *P* = 0.00047). Cutaneous microbiomes of RID that turned to chronic ulcer had significantly reduced bacterial diversity in comparison to either healthy individuals or rapidly healed RID (Kruskal Wallis: *P* = 0.00093).

**Figure 3:**
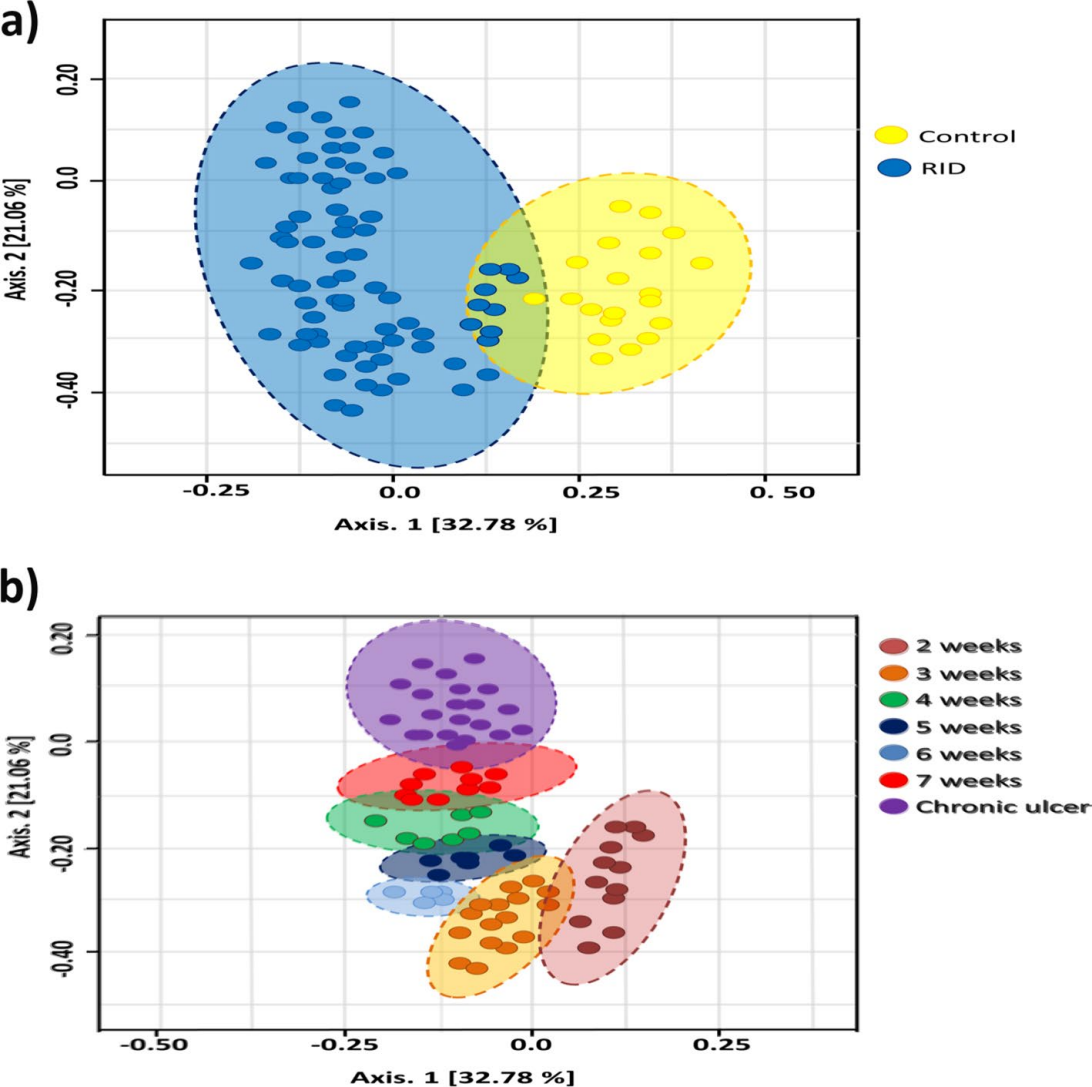
Principal coordinates analysis (PCoA) of cutaneous microbiomes based on weighted UniFrac distance matrices. PCoA plot represents the similarity distances between cutaneous microbiota of (**a**) healthy and diseased groups, (**b**) only RID microbiomes. Ellipses indicate the significant clustering (Adonis: r2 = 0.047, P = 0.00092) that was based on health state (**a**) and time required for healing (**b**).

Both structure and composition of bacterial communities significantly drove the classification of cutaneous microbiomes to health-state based clustering (Fig. 3a). PCoA based on weighted UniFrac metrics have sorted RID associated samples to 7 particular clusters that were mainly based on the healing time of RID (Fig. 3b).

### Relative proportions of core genera of healthy cutaneous microbiomes could potentially define the outcome of RID

**Figure 4:**
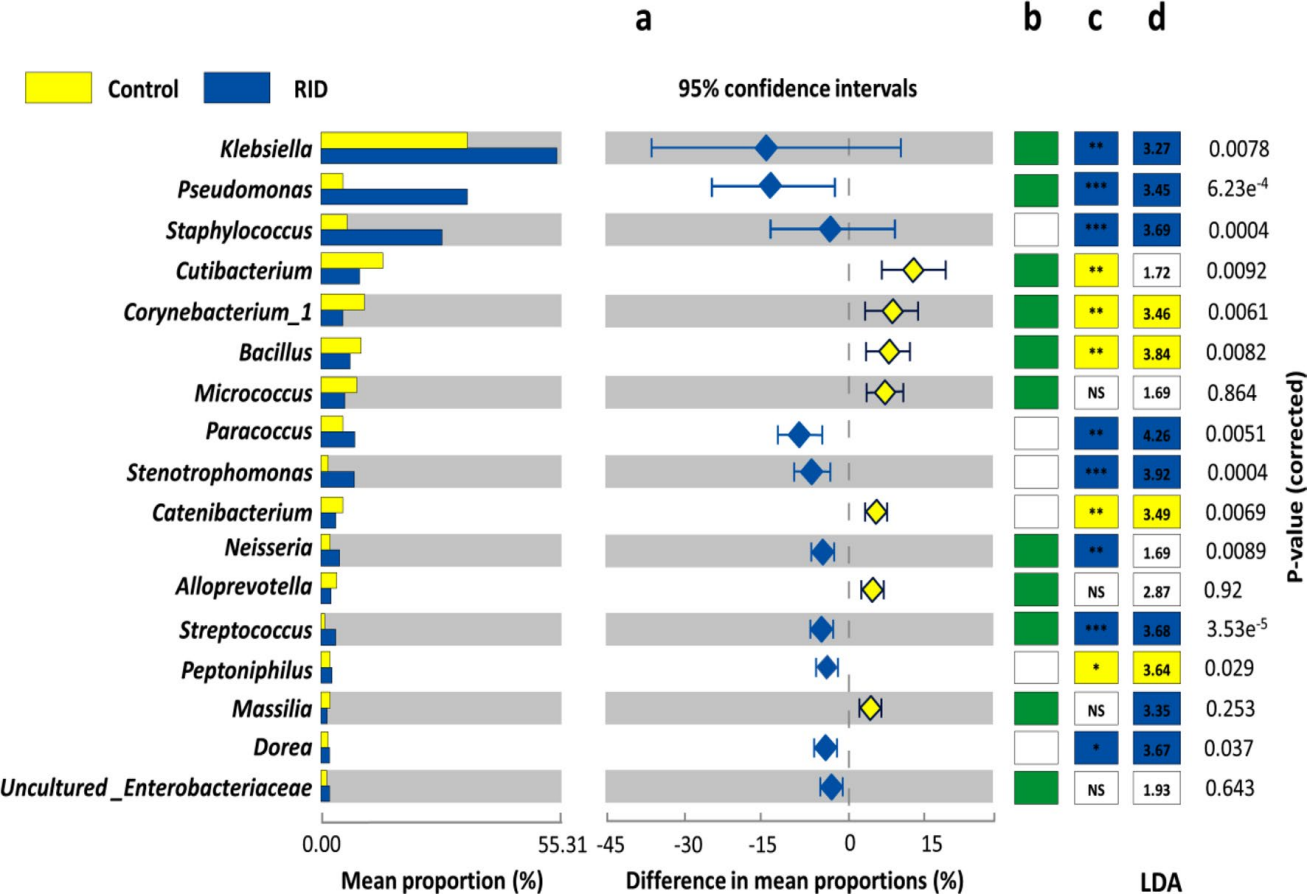
The relative abundance of the most predominant genera in cutaneous microbiomes of healthy and patients with RID groups. (**a**) Bar plots depict the mean proportion and differences in mean proportions with 95% confidence intervals. (**b**) The core genera of the entire dataset are indicated by green boxes. (**c**) Colored boxes indicate the core microbiome of healthy and patients with RID, and strikes represent the statistically different genera between study groups. Statistical significance of pairwise comparisons was defined using the nonparametric Wilcoxon rank-sum test. Significant differences were displayed with either ns, p-value > 1; *p < 0.05, **p < 0.01 or ***p < 0.001. (**d**) Potential biomarkers for each group were inferred using LEfSe and the numbers denote LDA scores.

Studying the structure of RID microbiomes at the family level unable to provide a deep understanding of the potential causative agent of RID. For instance, either *Enterobacteriaceae* or *Staphylococcaceae* were the most predominant families in several RID specimens, while genus level analysis was provided a clear separation of genera belonging to the previous families (Fig. 4a). In general, *Klebsiella*, *Cutibacterium*, *Corynebacterium*, *Bacillus* and *Paracoccus* were the dominant genera in healthy subjects. On the other hand, microbiomes of RID were generally inhabited with *Klebsiella*, *Staphylococcus* or *Pseudomonas*. Interestingly, microbiomes of RID that turned to chronic ulcers showed significant overrepresentation of *Klebsiella* or *Pseudomonas*, *Cutibacterium* and *Stenotrophomonas*. Also, the coexistence of *Pseudomonas*, *Staphylococcus* and *Stenotrophomonas* was significantly correlated with shifting of RID toward chronicity (Spearman; r = 0.87, *P* = 0.001).

Identification of the core genera of the entire dataset revealed that *Klebsiella*, *Pseudomonas*, *Cutibacterium*, *Corynebacterium*, *Bacillus* and uncultured *Enterobacteriaceae* were detected in all samples with variable abundance (Fig. 4b).

### Tendency toward the development of radiotherapy-induced chronic ulcers was significantly linked to Dermotype C

Our genera were stratified into three dermotypes (Table 2). Dermotype C was characterized by the predominance of *Pseudomonas*, *Staphylococcus* and *Stenotrophomonas* over *Klebsiella*. Of note, although dermotype A in healthy individuals was composed of slightly dominant *Klebsiella* in addition to *Bacillus*, *Cutibacterium* and *Staphylococcus*, it was characterized by an overrepresentation of *Klebsiella* in RID. Moreover, both dermotype A in RID and dermotype C were significantly linked to delayed recovery and chronic ulcer formation in RID patients (Table 3).

**Table 2:** Dermotypes that were detected in cutaneous microbiome.

Dermotype A	Dermotype B	Dermotype C
*Klebsiella*	*Staphylococcus*	*Pseudomonas*
*Bacillus*	*Cutibacterium*	*Staphylococcus*
*Cutibacterium*	*Klebsiella*	*Stenotrophomonas*
*Corynebacterium*	*Streptococcus*	*Cutibacterium*
*Acinetobacter*	*Pseudomonas*	*Paracoccus*
*Aloprevotella*	*Acinetobacter*	*Bacillus*
*Pseudomonas*	*Gemella*	*Klebsiella*
*Finegoldia*	*Massilia*	*Acinetobacter*

**Table 3:** Summary of case number in each dermotype.

Dermotype	Control (n = 20)	RID (Total) (n = 78)	2 weeks (n = 12)	3 weeks (n = 16)	4 weeks (n = 5)	5 weeks (n = 7)	6 weeks (n = 8)	7 weeks (n = 9)	Chronic (n = 21)
Dermotype A (N, %) ( Klebsiella-dominated)	8(40)	24(30.7)	1(8.3)	3(18.7)	0	2(28.5)	6(75)	3(33.3)	9(42.8)
B dermotype B (N, %) (Staphylococcus-dominated)	12(60)	32(41)	11(91.7)	13(81.3)	5(100)	1(14.3)	0	0	2(9.5)
Dermotype C (N, %) (Pseudomonas-dominated)	0	22(28.2)	0	0	0	4(57)	2(25)	6(66.7)	10(47.6)

### Predominance of *Klebsiella pneumoniae* or co-existence of Pseudomonas aeruginosa and *Staphylococcus aureus* was significantly linked to RID

Species level analysis of RSVs revealed the significant enrichment of *Klebsiella pneumoniae*, *Pseudomonas aeruginosa* and *Staphylococcus aureus* in RID in comparison to healthy subjects (Mean relative abundance: 48.94% and 27.54%, respectively; Wilcoxon test: *P* = 0.00017). Additionally, the predominance of these species was accompanied by a slightly enriched representation of uncultured *Enterobacteriaceae* species, *Klebsiella varicola*, *Stenotrophomonas maltophilia, Rothia dentocariosa* and *Acinetobacter johnsonii*. Nevertheless, unclassified sequences represented about 21.83% of cutaneous microbiomes of controls, while in RID microbiomes they were constituted 1.69%. Strikingly, *K. pneumoniae* was negatively correlated to *P. aeruginosa* in all study groups (Spearman: *r* = − 0.79; *P* = 0.0008). Also, the strongest correlations were detected between *P. aeruginosa*, *S. aureus* and *S. maltophilia*.

### Overall functional potential of the cutaneous microbiome was likely to contribute to the pathogenesis of RID

The collective metabolic functional profile of RID microbiome was significantly associated with the overrepresentation of metabolic pathways that could be attributed to impairments in epidermal integrity, alteration in pH and supporting microbial survival such as lipid metabolism, histidine metabolism, enhanced bacterial invasion of epithelial cells, signaling molecules of bacterial toxins, hedgehog signaling pathway, nitrogen metabolism and peptidases (Supplemental Fig. S1).

## Discussion

RID represents a challenge for clinicians, especially it is nearly acquired by all cancer patients who undergo radiotherapy and is regarded as an extra section in healthcare expenditure. Till now, a detailed characterization of microbial contribution to the bioburden of skin disorders is exclusively concerned with studying atopic dermatitis, psoriasis, diabetic foot and burns. Furthermore, the major concern is mainly directed to the culture-based approaches that are restricted to a scanty number of microorganisms, and consequentially override the integral role of the entire community^38,39^.

In accordance with the well-established concepts, concerning alpha diversity, about the evident reduction in the microbial diversity of dermatological disorders such as atopic dermatitis and psoriasis, the microbiome of RID showed minimal bacterial diversity in comparison to controls^21,40–43^. Also radiotherapy was accompanied by significant reduction in microbial diversity of salivary microbiome^44^. The pathophysiology of RID may include epidermal disturbances, cutaneous injuries, reduced blood supply, impairment in cellular repair and misdirected cutaneous immunity. These factors could be explaining the lowered diversity of RID as a result of predominance of certain opportunistic pathogens over the beneficial ones that subsequently interfere with the synthesis of natural antimicrobial peptides and cutaneous immunomodulation^45,46^. Another remarkable observation is the recovery rates of RID that were positively correlated to bacterial diversity at different taxonomy levels. These observations were matched with the protective roles of cutaneous microbiota in the maintenance of epidermal integrity^45^. The impact of restoring skin microbiome on wound healing has been revealed by a study on the animal model^47^.

It is noteworthy that the UniFrac-based similarity distances between RID microbiomes significantly assorted the specimens to particularly separate patterns depending on the recovery time. A recent study revealed the significant stable temporal shifts of nasopharyngeal microbiota during the period of radiotherapy treatment^48^. Thus, the composition and structure of microbiomes at beginning of RID could reflect the prospective outcome.

Taxonomy profiling of cutaneous microbiota associated to RID highlighted the massive prevalence of Proteobacteria or Firmicutes that is in agreement with the overrepresentation of taxa belonging to them in common skin disorders such acne, atopic dermatitis and psoriasis^21,43,49^.

One of the interesting findings is the significant association between diabetes mellitus and developing a chronic ulcer in RID patients (Spearman: r = 0.86; *P* = 0.001). Diabetes mellitus is a metabolic disorder that is significantly linked to developing chronic wounds due to the impairments in wound healing phases that provides favorable conditions for either opportunistic residents or pathogenic bacteria^50^. Also, colonization of wounds with certain microorganisms was strongly correlated to the persistence of wound via organism induced body changes that include epidermal barrier integrity, alteration in pH and dysregulation of the immune system^51–53^. Overall, developing chronic ulcer in diabetic patients with RID could potentially depend on the colonizing microorganisms through intensifying the epidermal disturbances produced by diabetes mellitus.

The main interesting finding of the present study is the collective correlations between community members. While *Klebsiella* genus was inversely correlated to other predominant genera in all datasets, *Pseudomonas*, *Staphylococcus* and *Stenotrophomonas* have exhibited a significant coexistence especially in prolonged RID and chronic ulcers (Supplemental Table S3). Although the impact of coinfection with *S. aureus* and *P. aeruginosa* on the clinical outcome of cystic fibrosis is controversial, the comparisons with mono-infection of *S. aureus* or *P. aeruginosa* showed that the pair coexisting was significantly associated with prolonged hospitalization and exacerbation of the disease^54^. Also, the bidirectional interplay between this pair was manifested in the inhibition of phagocytosis, in addition to the promotion of staphylococcal biofilm formation by *P. aeruginosa*^54,55^. Additionally, *Stenotrophomonas maltophilia* was detected in our samples with significant prevalence especially in RID patients. *S. maltophilia* has emerged as an opportunistic pathogen with global concerns, due to its significant association with the high crude mortality in pneumonia and bacteremia of immunosuppressed patients^56^. *S. maltophilia* was also reported to cause wound infections, soft tissue infections, bone and joint infections, and cellulitis^57,58^. Interestingly, in our study, the coexistence of *S. maltophilia* and *P. aeruginosa* was strongly correlated to develop chronic ulcers in RID. This observation was in agreement with the previously reported cooperative pathogenicity of this pair in cystic fibrosis^59^.

As expected in the cutaneous microbiome, *Staphylococcus* genus was defined as a core genus in healthy and diseased groups. Although *S. aureus* was significantly enriched in RID microbiomes, it was overrepresented in the rapidly healed RID. These findings were matched with the previous study which reported that the *S. aureus* infection was conjugated with limited exacerbations and a short duration of hospitalization^54^.

Notably, *K. pneumoniae* showed fluctuated representation in our specimens. In microbiomes of RID, *K. pneumoniae* was significantly overrepresented especially in samples that turned to a chronic ulcer, and its abundance was strongly linked to diabetes mellitus. As it is known that *K. pneumoniae* have been implicated in liver abscess, meningitis and psoas abscess^60^. Furthermore, *K. pneumoniae* may harbor diverse virulence characteristics including capsules, multidrug resistance and virulence factors that enhance invasion and promote epidermal deteriorations^61,62^. The coexistence of these characteristics and diabetes mellitus could boost epidermal damage, aggravate the inflammatory processes and retard the repair of the epidermal barrier.

The limitations of this study are the slightly small sample size of each group, dependence on unculturable approach, lack of microbiome characterization at different sampling time and isolation of causative agents in order to define their virulence characteristics including antimicrobial resistance patterns.

## Conclusions

We have studied the microbiome of patients with RID in order to accentuate the underlying bacterial structure and to provide new insight about the potential role of microbiota as an influential factor in pathogenesis and clinical outcome of RID as well. A notable positive correlation has existed between bacterial diversity and recovery rates of RID. The overwhelming predominance of *Klebsiella* or coexistence of *Pseudomonas*, *Staphylococcus* and *Stenotrophomonas* potentially prompt the chronic ulcer formation in RID patients. Cutaneous microbiome profiling at the early stages of RID could be indicative of the prospective clinical outcomes and might be a helpful guide for personalized therapy and management of RID. Finally, shifts in the structure and prevalence of cutaneous microbiota that accompany the RID could support the candidate role of microbial dysbiosis in the pathogenesis of RID.

## Supplementary Information

Supplementary Information.
