# Supplementary material for: Alterations in skin microbiome mediated by radiotherapy and their potential roles in the prognosis of radiotherapy-induced dermatitis: a pilot study
Source: Sci Rep. 2021 Mar 4;11:5179. doi: 10.1038/s41598-021-84529-7 (PMC7933139; doi:10.1038/s41598-021-84529-7)
Supplement: Supplementary file 1 — Supplementary Information. [file 41598_2021_84529_MOESM1_ESM.docx]

**Alterations in skin microbiome mediated by radiotherapy and their potential roles in the prognosis of radiotherapy-induced dermatitis: a pilot study**

**Mohammed Ramadan ^1^, Helal F. Hetta^2,3^*, Moustafa M. Saleh^4^, Mohamed E. Ali^1^, Ali Aya Ahmed^5^, and Mohammed Salah^4^**

**^1^** Microbiology and Immunology Department, Faculty of Pharmacy Al-Azhar University-Assiut branch, Assiut, 71526, Egypt.

^2^ Department of Medical Microbiology and Immunology, Faculty of Medicine, Assiut University, Assiut, 71515, Egypt

^3^Department of Internal Medicine, University of Cincinnati College of Medicine, Cincinnati, OH 45267-0595, USA.

**^4^** Microbiology and Immunology Department, Faculty of Pharmacy Port Said University, Port Said, 42526, Egypt.

**^5^** Microbiology and Immunology Department, Faculty of Pharmacy Sinai University, Ismaillia, 41611, Egypt.

***Correspondence:**

**Helal F. Hetta,**

Department of Medical Microbiology and Immunology, Faculty of Medicine,

Assiut University, Assiut, 71515, Egypt. [**helalhetta@aun.edu.eg**](mailto:helalhetta@aun.edu.eg)

Department of Internal Medicine, University of Cincinnati College of Medicine,

Cincinnati, OH 45267-0595, USA.

[**helal.hetta@uc.edu**](mailto:helal.hetta@uc.edu)

<https://orcid.org/0000-0001-8541-7304>

**Table S1: Demographic data and clinical characteristics of the individuals enrolled in addition to dermotype.**

| **Patient ID** | **Age** | **Sex** | **Cancer** | **Site of RID** | **Time of RID appearance**  **(Number of sessions)*** | **Other diseases** | **Enterotype** | **Outcome** |
| --- | --- | --- | --- | --- | --- | --- | --- | --- |
| 1 | 38 | F | Breast | Chest | 5 | No | C | Recovered after 7 weeks |
| 2 | 51 | F | Breast | Chest | 8 | DM | C | Chronic ulcer |
| 3 | 65 | M | Prostate | Sacrum | 9 | Hypertension | C | Recovered after 7 weeks |
| 4 | 38 | M | Colorectal | Pelvis | 5 | DM | A | Recovered after 5 weeks |
| 5 | 53 | M | Lung | Chest | 7 | DM  Hypertension | A | Recovered after 5 weeks |
| 6 | 65 | M | Lymphoma | Leg | 3 | No | B | Recovered after 3 weeks |
| 7 | 49 | F | Lung | Chest | 6 | No | A | Chronic ulcer |
| 8 | 41 | F | Brain | Head | 4 | No | B | Recovered after 3 weeks |
| 9 | 51 | F | Breast | Sacrum | 7 | DM | B | Recovered after 4 weeks |
| 10 | 54 | M | Lung | Chest | 2 | DM | C | Chronic ulcer |
| 11 | 47 | M | Cervical | Pelvis | 3 | No | B | Recovered after 3 weeks |
| 12 | 42 | M | Prostate | Sacrum | 7 | No | B | Recovered after 3 weeks |
| 13 | 38 | F | Cervical | Pelvis | 5 | Hypertension | B | Recovered after 3 weeks |
| 14 | 42 | F | Breast | Chest | 5 | NO | B | Recovered after 3 weeks |
| 15 | 49 | F | Breast | Chest | 7 | DM  Hypertension | B | Recovered after 2 weeks |
| 16 | 56 | F | Breast | Chest | 9 | DM | C | Chronic ulcer |
| 17 | 52 | M | Oropharyngeal | Neck | 4 | Hypertension | A | Recovered after 3 weeks |
| 18 | 43 | F | Breast | Chest | 10 | DM | C | Recovered after 7 weeks |
| 19 | 67 | M | Lung | Chest | 4 | No | B | Recovered after 4 weeks |
| 20 | 54 | F | Breast | Chest | 5 | No | B | Recovered after 3 weeks |
| 21 | 58 | F | Breast | Chest | 6 | DM | C | Chronic ulcer |
| 22 | 67 | M | Prostate | Sacrum | 9 | Hypertension | B | Recovered after 2 weeks |
| 23 | 32 | M | Colorectal | Pelvis | 7 | DM | A | Recovered after 7 weeks |
| 24 | 57 | M | Lung | Chest | 10 | No  Hypertension | A | Recovered after 2 weeks |
| 25 | 53 | M | Lymphoma | Leg | 5 | No | A | Chronic ulcer |
| 26 | 47 | F | Breast | Chest | 12 | DM  Hypertension | A | Recovered after 6 weeks |
| 27 | 53 | F | Breast | Chest | 5 | DM | A | Chronic ulcer |
| 28 | 46 | M | Oropharyngeal | Neck | 4 | No | C | Chronic ulcer |
| 29 | 45 | F | Breast | Chest | 9 | DM | A | Recovered after 6 weeks |
| 30 | 63 | M | Lung | Chest | 4 | No | A | Recovered after 6 weeks |
| 31 | 54 | F | Cervical | Chest | 6 | No | C | Chronic ulcer |
| 32 | 38 | M | Brain | Head | 4 | DM | C | Recovered after 7 weeks |
| 33 | 53 | M | Colorectal | Sacrum | 6 | No | B | Recovered after 2 weeks |
| 34 | 35 | F | Colorectal | Pelvis | 5 | DM | A | Chronic ulcer |
| 35 | 64 | M | Prostate | Sacrum | 8 | No | B | Recovered after 2 weeks |
| 36 | 41 | F | Cervical | Pelvis | 4 | Hypertension | B | Recovered after 3 weeks |
| 37 | 56 | F | Breast | Chest | 6 | NO | A | Recovered after 7 weeks |
| 38 | 49 | F | Colorectal | Chest | 11 | DM  Hypertension | A | Recovered after 6 weeks |
| 39 | 56 | M | Prostate | Chest | 5 | DM | A | Chronic ulcer |
| 40 | 55 | M | Oropharyngeal | Neck | 3 | No | B | Recovered after 3 weeks |
| 41 | 49 | F | Breast | Sacrum | 6 | DM | B | Recovered after 4 weeks |
| 42 | 45 | M | Colorectal | Chest | 4 | DM | B | Chronic ulcer |
| 43 | 37 | F | Colorectal | Pelvis | 7 | No | B | Recovered after 2 weeks |
| 44 | 49 | M | Prostate | Sacrum | 10 | DM | B | Chronic ulcer |
| 45 | 46 | F | Cervical | Pelvis | 5 | Hypertension | A | Recovered after 3 weeks |
| 46 | 54 | M | Colorectal | Pelvis | 8 | DM | C | Recovered after 7 weeks |
| 47 | 47 | M | Lung | Chest | 8 | DM  Hypertension | B | Recovered after 2 weeks |
| 48 | 63 | F | Breast | Chest | 9 | Hypertension | B | Recovered after 3 weeks |
| 49 | 38 | F | Brain | Head | 4 | No | C | Recovered after 5 weeks |
| 50 | 48 | F | Breast | Sacrum | 12 | DM | B | Recovered after 4 weeks |
| 51 | 47 | M | Lung | chest | 5 | DM | C | Chronic ulcer |
| 52 | 32 | F | Colorectal | Pelvis | 3 | No | B | Recovered after 2 weeks |
| 53 | 57 | M | Prostate | Sacrum | 8 | DM | C | Chronic ulcer |
| 54 | 49 | F | Cervical | Pelvis | 5 | Hypertension | B | Recovered after 3 weeks |
| 55 | 56 | F | Breast | Chest | 7 | DM  Hypertension | A | Recovered after 6 weeks |
| 56 | 45 | F | Breast | Chest | 9 | NO | B | Recovered after 2 weeks |
| 57 | 49 | F | Breast | Chest | 10 | DM  Hypertension | C | Recovered after 6 weeks |
| 58 | 46 | F | Breast | Chest | 6 | DM | C | Chronic ulcer |
| 59 | 32 | M | Oropharyngeal | Neck | 7 | No | B | Recovered after 3 weeks |
| 60 | 42 | F | Breast | Chest | 9 | NO | A | Recovered after 3 weeks |
| 61 | 47 | F | Breast | Chest | 7 | DM | A | Recovered after 6 weeks |
| 62 | 47 | M | Colorectal | Chest | 4 | No | C | Recovered after 5 weeks |
| 63 | 43 | F | Breast | Chest | 6 | DM | C | Recovered after 7 weeks |
| 64 | 57 | F | Breast | Chest | 8 | DM | A | Chronic ulcer |
| 65 | 59 | M | Prostate | Sacrum | 6 | Hypertension | B | Recovered after 5 weeks |
| 66 | 52 | F | Breast | Sacrum | 8 | DM | B | Recovered after 4 weeks |
| 67 | 45 | M | Lung | chest | 4 | DM | A | Chronic ulcer |
| 68 | 40 | F | Brain | Head | 5 | No | B | Recovered after 2 weeks |
| 69 | 58 | F | Lung | Chest | 11 | No | A | Chronic ulcer |
| 70 | 38 | F | Brain | Head | 5 | No | B | Recovered after 2 weeks |
| 71 | 67 | M | Lung | Chest | 7 | Hypertension | C | Recovered after 5 weeks |
| 72 | 38 | F | Breast | Chest | 5 | DM | C | Recovered after 7 weeks |
| 73 | 48 | F | Breast | Chest | 7 | DM | A | Chronic ulcer |
| 74 | 62 | M | Prostate | Sacrum | 6 | Hypertension | B | Recovered after 3 weeks |
| 75 | 58 | M | Colorectal | Pelvis | 5 | No | A | Recovered after 7 weeks |
| 76 | 47 | M | Lung | Chest | 7 | DM  Hypertension | C | Recovered after 5 weeks |
| 77 | 57 | M | Lymphoma | Leg | 5 | Hypertension | B | Recovered after 2 weeks |
| 78 | 54 | F | Lung | Chest | 9 | No | C | Chronic ulcer |

* = Radiotherapy sessions are usually five days a week, DM = Diabetes mellitus

**Table S2: Mean relative abundance of the major phyla (≥0.1)**

| Phylum | Control | RID  (Total) | 2 weeks | 3 weeks | 4 weeks | 5 weeks | 6 weeks | 7 weeks | Chronic |
| --- | --- | --- | --- | --- | --- | --- | --- | --- | --- |
| Epsilonbacteraeota | 0.192 | 0.181 | 0.100 | 0.331 | 0.460 | 0.100 | 0.100 | 0.108 | 0.128 |
| Tenericutes | 0.200 | 0.299 | 0.200 | 0.475 | 0.720 | 0.200 | 0.200 | 0.206 | 0.200 |
| TM7 | 0.300 | 0.353 | 0.300 | 0.431 | 0.580 | 0.300 | 0.300 | 0.300 | 0.300 |
| Cyanonbacteria | 0.400 | 0.444 | 0.400 | 0.513 | 0.640 | 0.400 | 0.456 | 0.400 | 0.400 |
| Fusobacteria | 1.510 | 0.723 | 1.091 | 0.822 | 0.835 | 0.058 | 0.999 | 0.455 | 0.518 |
| Bacteroidetes | 3.266 | 3.010 | 4.422 | 4.525 | 4.133 | 2.096 | 1.939 | 1.453 | 1.751 |
| Actinobacteria | 9.325 | 7.149 | 12.080 | 12.327 | 7.094 | 5.247 | 3.324 | 3.405 | 4.357 |
| Proteobacteria | 40.896 | 55.388 | 31.035 | 29.438 | 25.212 | 64.037 | 80.925 | 80.619 | 74.009 |
| Firmicutes | 43.912 | 32.453 | 50.154 | 51.138 | 60.325 | 27.561 | 11.757 | 13.068 | 18.365 |
| *Proteobacteria/Firmicutes | 0.968 | 4.094 | 0.645 | 0.607 | 0.430 | 2.564 | 8.041 | 7.211 | 7.280 |

* Proteobacteria/ Firmicutes ratio of all samples of each group

**Table S3: Correlations between the top 25 genera in all samples based on Spearman correlation coefficient.**

|  | Klebsiella | Bacillus | Pseudomonas | Cutibacterium | Stenotrophomonas | Corynebacterium | Staphylococcus | Acinetobacter | Paracoccus | Rothia | Micrococcus | Neisseria | Alloprevotella | peptoniphilus | Massilia | Dorea | Streptococcus | Selenomonas | Sphingomonas | Anaerococcus | Cronobacter | Stenotrophomonas | Enhydrobacter | Erwinia | Finegoldia |
| --- | --- | --- | --- | --- | --- | --- | --- | --- | --- | --- | --- | --- | --- | --- | --- | --- | --- | --- | --- | --- | --- | --- | --- | --- | --- |
| Klebsiella | 1.00 | -0.31 | -0.76 | -0.32 | -0.84 | 0.31 | -0.89 | -0.65 | 0.37 | -0.53 | -0.88 | -0.55 | 0.39 | 0.67 | 0.54 | -0.35 | -0.66 | 0.69 | -0.39 | 0.41 | 0.64 | 1.00 | 0.84 | 0.86 | 0.52 |
| Bacillus | -0.31 | 1.00 | -0.50 | -0.50 | 0.77 | -0.31 | 0.87 | -0.44 | 0.42 | 1.00 | 0.43 | 0.69 | 0.55 | 0.58 | 0.56 | 0.74 | 0.48 | 0.53 | 0.87 | 0.70 | 0.61 | -0.34 | 0.50 | 0.67 | 0.99 |
| Pseudomonas | -0.76 | -0.50 | 1.00 | 0.68 | 0.96 | 0.48 | 0.92 | -0.31 | 0.54 | 0.99 | 0.70 | 0.75 | 0.80 | -0.31 | 0.39 | 0.30 | 0.66 | 1.00 | 0.94 | 0.44 | 0.49 | -0.51 | 0.84 | 0.72 | -0.33 |
| Cutibacterium | -0.32 | -0.50 | 0.68 | 1.00 | 0.69 | 0.40 | 0.67 | -0.46 | 0.32 | -0.34 | 0.43 | 0.48 | 0.56 | 0.65 | 0.66 | 0.76 | 0.65 | 0.88 | 0.35 | -0.54 | 0.53 | 1.00 | 0.37 | -0.48 | 0.69 |
| Stenotrophomonas | -0.84 | 0.77 | 0.96 | 0.69 | 1.00 | 0.85 | 0.89 | 0.66 | 0.35 | -0.52 | 0.87 | 0.90 | 0.33 | 0.66 | 0.59 | 0.58 | 0.73 | 0.85 | 0.58 | 0.69 | 0.87 | 0.39 | 0.36 | 0.49 | 0.85 |
| Corynebacterium | 0.31 | -0.31 | 0.48 | 0.40 | 0.85 | 1.00 | 0.86 | 0.66 | 0.38 | 0.99 | 1.00 | -0.55 | 0.32 | 0.53 | 0.32 | 0.80 | 0.78 | 0.66 | 0.42 | 0.65 | 0.85 | 0.89 | 0.70 | 0.80 | 0.76 |
| Staphylococcus | -0.89 | 0.87 | 0.92 | 0.67 | 0.90 | 0.86 | 1.00 | 0.43 | 0.56 | -0.31 | 0.89 | 0.70 | 0.98 | 0.51 | -0.45 | -0.37 | 0.40 | 0.70 | 0.69 | 0.71 | 0.66 | 0.58 | 0.43 | 0.74 | 0.56 |
| Acinetobacter | -0.65 | -0.44 | -0.31 | -0.46 | 0.66 | 0.66 | 0.43 | 1.00 | 0.47 | 0.88 | 0.58 | 0.53 | 0.56 | 0.57 | 0.58 | 0.33 | 0.72 | 0.71 | 0.37 | 0.53 | 0.70 | 0.88 | 0.83 | 0.48 | 0.42 |
| Paracoccus | 0.37 | 0.42 | 0.54 | 0.32 | 0.35 | 0.38 | 0.56 | 0.47 | 1.00 | 0.93 | 0.88 | 0.36 | -0.50 | 0.32 | -0.39 | -0.36 | 0.40 | 0.90 | 0.42 | -0.52 | 0.71 | 0.60 | 0.66 | -0.60 | 0.88 |
| Rothia | -0.53 | 1.00 | 0.99 | -0.34 | -0.52 | 0.99 | -0.31 | 0.88 | 0.93 | 1.00 | 0.60 | 0.50 | 0.46 | 0.61 | 0.52 | 0.68 | 0.69 | 0.53 | 0.54 | 0.43 | 0.89 | 0.89 | -0.45 | 0.70 | 0.88 |
| Micrococcus | -0.88 | 0.43 | 0.70 | 0.43 | 0.87 | 1.00 | 0.89 | 0.58 | 0.88 | 0.60 | 1.00 | 0.47 | 0.38 | 0.32 | 0.54 | 0.63 | 0.66 | 0.34 | 0.32 | 0.35 | 0.54 | 0.45 | 0.82 | -0.37 | 0.65 |
| Neisseria | -0.55 | 0.69 | 0.75 | 0.48 | 0.90 | -0.55 | 0.70 | 0.53 | 0.36 | 0.50 | 0.47 | 1.00 | -0.50 | 0.39 | 0.56 | 0.44 | 0.37 | 0.69 | 0.35 | -0.52 | 0.34 | 0.32 | 0.83 | 0.65 | 0.98 |
| Alloprevotella | 0.39 | 0.55 | 0.80 | 0.56 | 0.33 | 0.32 | 0.98 | 0.56 | -0.50 | 0.46 | 0.38 | -0.50 | 1.00 | 0.55 | 0.35 | -0.35 | 0.69 | 0.62 | 0.38 | 0.71 | 0.69 | 0.70 | 0.94 | 0.64 | 0.66 |
| Peptoniphilus | 0.67 | 0.58 | -0.31 | 0.65 | 0.66 | 0.53 | 0.51 | 0.57 | 0.32 | 0.61 | 0.32 | 0.39 | 0.55 | 1.00 | 0.33 | 0.60 | 0.54 | 0.90 | 0.56 | 0.75 | 0.62 | 0.49 | -0.35 | 0.33 | 0.32 |
| Massilia | 0.54 | 0.56 | 0.39 | 0.66 | 0.59 | 0.32 | -0.45 | 0.58 | -0.39 | 0.52 | 0.54 | 0.56 | 0.35 | 0.33 | 1.00 | 0.57 | -0.39 | 0.62 | 0.47 | 0.62 | 0.90 | 0.88 | -0.51 | 0.72 | 0.80 |
| Dorea | -0.35 | 0.74 | 0.30 | 0.76 | 0.58 | 0.80 | -0.37 | 0.33 | -0.36 | 0.68 | 0.63 | 0.44 | -0.35 | 0.60 | 0.57 | 1.00 | 0.67 | -0.56 | -0.32 | 0.99 | 0.61 | 0.68 | 1.00 | 0.63 | 0.72 |
| Streptococcus | -0.66 | 0.48 | 0.66 | 0.65 | 0.73 | 0.78 | 0.40 | 0.72 | 0.40 | 0.69 | 0.66 | 0.37 | 0.69 | 0.54 | -0.39 | 0.67 | 1.00 | 0.92 | 0.52 | -0.33 | -0.56 | -0.50 | -0.30 | 0.70 | 0.66 |
| Selenomonas | 0.69 | 0.53 | 1.00 | 0.88 | 0.85 | 0.66 | 0.70 | 0.71 | 0.90 | 0.53 | 0.34 | 0.69 | 0.62 | 0.90 | 0.62 | -0.56 | 0.92 | 1.00 | 0.58 | 0.31 | 0.92 | 0.87 | 0.40 | 0.55 | 0.37 |
| Sphingomonas | -0.39 | 0.87 | 0.94 | 0.35 | 0.58 | 0.42 | 0.69 | 0.37 | 0.42 | 0.54 | 0.32 | 0.35 | 0.38 | 0.56 | 0.47 | -0.32 | 0.52 | 0.58 | 1.00 | -0.52 | -0.38 | -0.36 | 0.86 | -0.36 | 0.63 |
| Anaerococcus | 0.41 | 0.70 | 0.44 | -0.54 | 0.69 | 0.65 | 0.71 | 0.53 | -0.52 | 0.43 | 0.35 | -0.52 | 0.71 | 0.75 | 0.62 | 0.99 | -0.33 | 0.31 | -0.52 | 1.00 | 0.87 | 0.87 | 0.67 | 0.70 | 0.49 |
| Cronobacter | 0.64 | 0.61 | 0.49 | 0.53 | 0.87 | 0.85 | 0.66 | 0.70 | 0.71 | 0.89 | 0.54 | 0.34 | 0.69 | 0.62 | 0.90 | 0.61 | -0.56 | 0.92 | -0.38 | 0.87 | 1.00 | 0.94 | 0.72 | 0.68 | 1.00 |
| Uncultured_enterobacreiace | 1.00 | -0.34 | -0.51 | 1.00 | 0.39 | 0.89 | 0.58 | 0.88 | 0.60 | 0.89 | 0.45 | 0.32 | 0.70 | 0.49 | 0.88 | 0.68 | -0.50 | 0.87 | -0.36 | 0.87 | 0.94 | 1.00 | -0.48 | 0.82 | 0.84 |
| Enhydrobacter | 0.84 | 0.50 | 0.84 | 0.37 | 0.36 | 0.70 | 0.43 | 0.83 | 0.66 | -0.45 | 0.82 | 0.83 | 0.94 | -0.35 | -0.51 | 1.00 | -0.30 | 0.40 | 0.86 | 0.67 | 0.72 | -0.48 | 1.00 | 0.63 | 0.74 |
| Erwinia | 0.86 | 0.67 | 0.72 | -0.48 | 0.49 | 0.80 | 0.74 | 0.48 | -0.60 | 0.70 | -0.37 | 0.65 | 0.64 | 0.33 | 0.72 | 0.63 | 0.70 | 0.55 | -0.36 | 0.70 | 0.68 | 0.82 | 0.63 | 1.00 | 0.70 |
| Finegoldia | 0.52 | 0.99 | -0.33 | 0.69 | 0.85 | 0.76 | 0.56 | 0.42 | 0.88 | 0.88 | 0.65 | 0.98 | 0.66 | 0.32 | 0.80 | 0.72 | 0.66 | 0.37 | 0.63 | 0.49 | 1.00 | 0.84 | 0.74 | 0.70 | 1.00 |


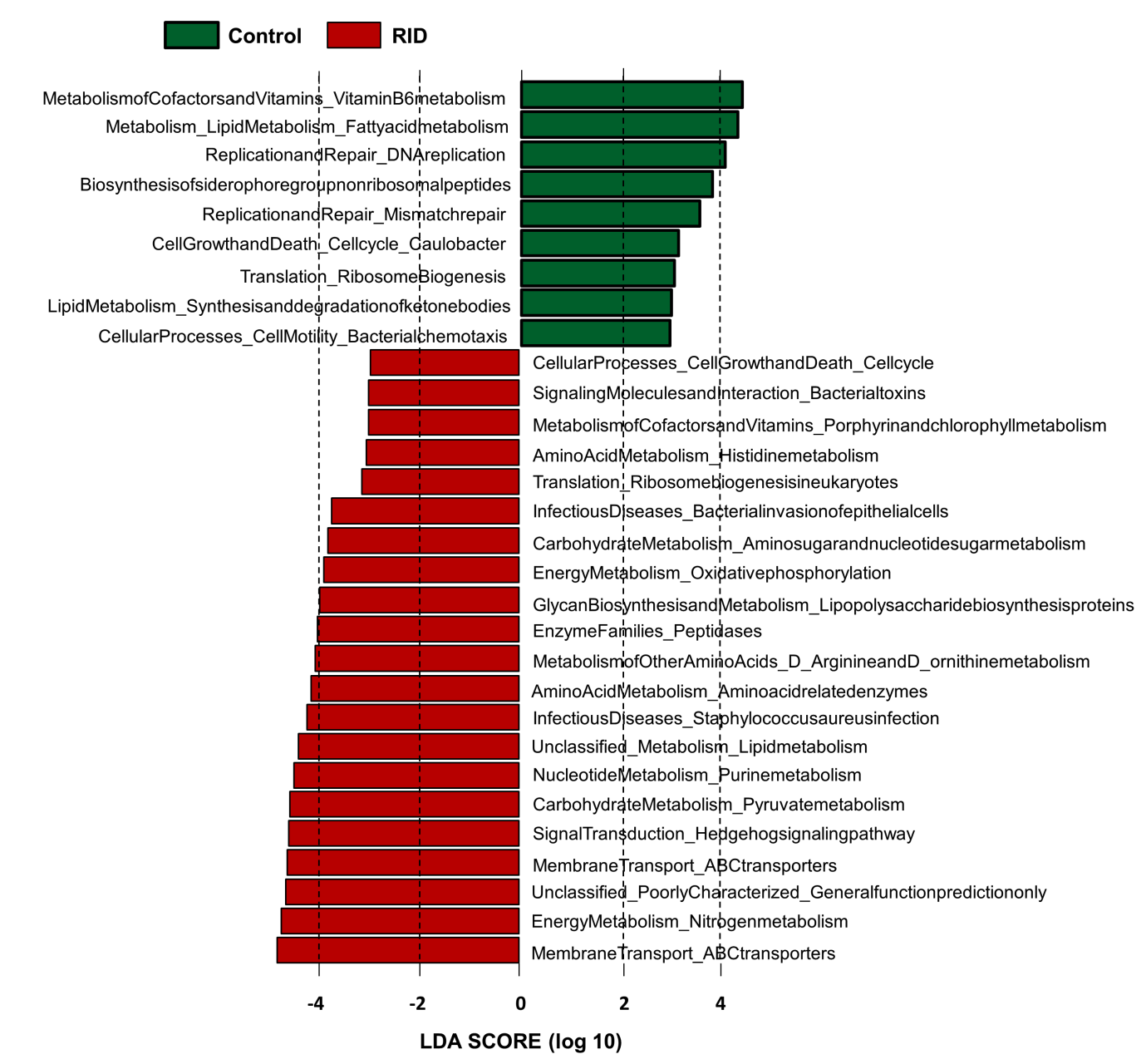


**Figure S1.** LEfSe analysis of differentially abundant metabolic pathways between healthy and patients with RID groups (https://huttenhower.sph.harvard.edu/lefse/) ^1^.

**References**

1 Segata, N. *et al.* Metagenomic biomarker discovery and explanation. *Genome biology* **12**, R60-R60, DOI: <https://doi.org/10.1186/gb-2011-12-6-r60> (2011).
